# The BCL2 inhibitor ABT-199 significantly enhances imatinib-induced cell death in chronic myeloid leukemia progenitors

**DOI:** 10.18632/oncotarget.1925

**Published:** 2014-04-27

**Authors:** Tun Kiat Ko, Charles T.H. Chuah, John W.J. Huang, King-Pan Ng, S. Tiong Ong

**Affiliations:** ^1^ Cancer and Stem Cell Biology Program, Duke-NUS Graduate Medical School, Singapore; ^2^ Department of Haematology, Singapore General Hospital, Singapore; ^3^ Department of Medical Oncology, National Cancer Centre, Singapore; ^4^ Department of Medicine, Duke University Medical Center, Durham, NC

**Keywords:** ABT-199, BH3 mimetic, imatinib, CML, normal cord blood, progenitors

## Abstract

BCR-ABL1-specific tyrosine kinase inhibitors prolong the life of patients with chronic myeloid leukemia (CML) but cannot completely eradicate CML progenitors. The BH3 mimetic, ABT-263, targets prosurvival BCL2 family members, and has activity against CML progenitors. However, the inhibitory effect of ABT-263 on BCL-X_L_, which mediates platelet survival, produces dose-limiting thrombocytopenia. A second-generation BH3 mimetic, ABT-199, has been developed to specifically bind BCL2 but not BCL-X_L_. We determined the activity of ABT-199 against CML cell lines, as well as primary CML and normal cord blood (NCB) progenitors. We find that BCL2 expression levels predict sensitivity to ABT-199 in CML and NCB progenitors, and that high NCB BCL2 levels may explain the reported hematologic toxicities in ABT-199-treated patients. Also, while single agent ABT-199 has modest activity against CML progenitors, when combined with imatinib, ABT-199 significantly enhances imatinib activity against CML progenitors at concentrations predicted to avoid hematologic toxicities.

## INTRODUCTION

Tyrosine kinase inhibitors (TKIs) that specifically target BCR-ABL11 have significantly prolonged the life of patients with chronic myeloid leukemia (CML) [1]. However, TKIs do not completely eliminate CML progenitors, and this may potentially result in disease relapse [2, 3]. Therefore, there is an ongoing search for therapeutic strategies that can completely eradicate CML progenitors. Recent studies have highlighted an important role for the pro-survival BCL2 protein in myeloid leukemia stem cell survival and maintenance [4, 5]. Consistent with these reports, we and others have previously shown that the BH3 mimetics, ABT-737 and ABT-263 which target BCL2 family members, can significantly enhance TKI-induced cell death in primary CML cells [6-9]. ABT-737 and ABT-263 have broad specificity against anti-apoptotic BCL2 family members [10, 11]. However, in clinical trials that employ ABT-263, a dose-limiting toxicity is thrombocytopenia [12]. This is due to the inhibitory effect of ABT-263 on BCL-X_L_, which is pivotal for platelet survival [13, 14]. Recently, a new BH3 mimetic, ABT-199, has been developed to selectively bind BCL2 but not BCL-X_L_, and thus does not harm platelets [15]. We therefore evaluated the activity of ABT-199 against CML progenitors.

## RESULTS

**Figure 1:**
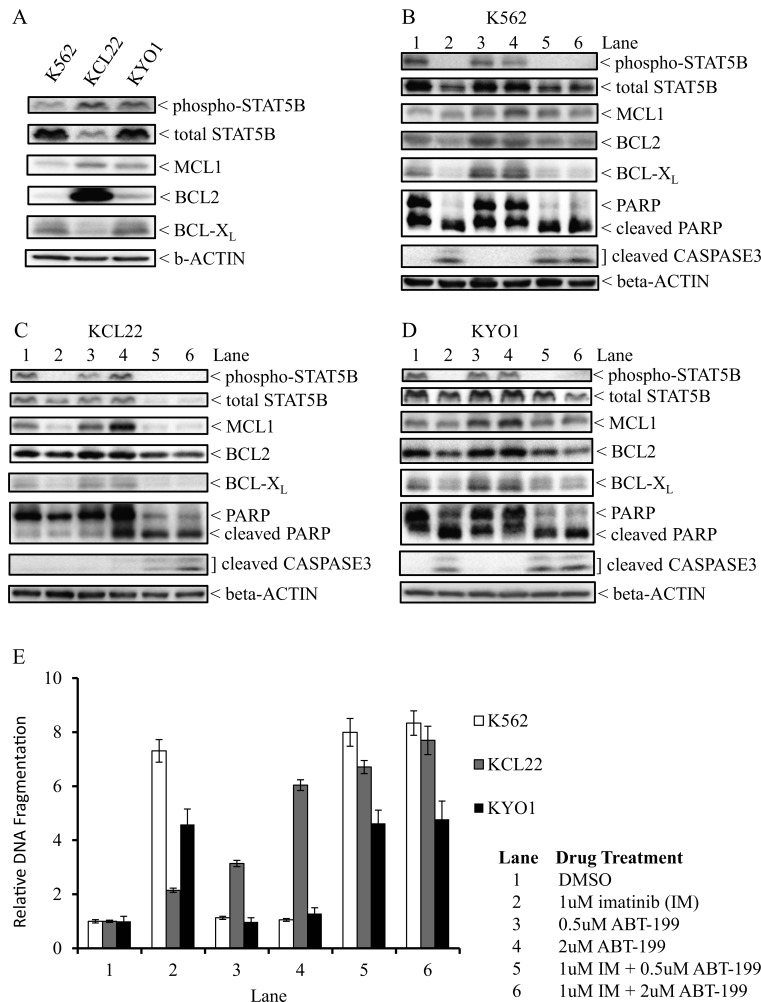
The effects of ABT-199 on the viability of CML cell lines (A) Western analysis of the protein expression levels of BCL2, BCL-X_L_ and MCL1 in selected CML cell lines. The phosphorylation state of STAT5B is a surrogate marker for BCR-ABL1 activity. Western analyses on the effects of ABT-199, alone or in combination with 2uM imatinib, on (B) K562, (C) KCL22, and (D) KYO1 cells. The cells underwent different drug treatments for 48 hours before they were harvested. (E) ELISA-based DNA fragmentation assay for the three CML cell lines that underwent different drug treatments for 48 hours before they were analysed. DNA fragmentation is calculated as a ratio of the reading for a given sample to that of the DMSO control. Results are given as the mean +/− s.e.m. (n=3). The drug treatment for each lane is indicated in the figure.

We first assessed the ability of ABT-199 to induce apoptosis in three CML cell lines, K562, KCL22, and KYO1, and correlated ABT-199 sensitivity to the protein expression level of pro-survival BCL2 family members. Here, we focused on BCL2 family members that promote CML cell survival in a BCR-ABL1-dependent fashion, notably BCL2, BCL-XL and MCL1 [16-18]. First, we found that BCL2 expression levels was at least 5-fold higher in KCL22 cells compared to K562 and KYO1, and that both K562 and KYO1 expressed higher amounts of BCL-XL than KCL22, while for MCL1, both KCL22 and KYO1 expressed more than K562 (Figure 1A). In K562 and KYO1 cells, we found imatinib alone but not ABT-199 alone, resulted in decreased expression of all three pro-survival proteins, and that this was associated with the induction of apoptosis (Figures 1B-E). In KCL22 cells, imatinib exposure also resulted in decreased expression of all three BCL2 family members, but did not induce cell death, a finding that is consistent with the reported inability of these cells to activate cell death (Figure 1C, E) [7, 19]. However, and in contrast to K562 and KYO1 cells, KCL22 cells exhibited single agent-sensitivity to ABT-199 alone, which induced apoptosis in a dose-dependent manner (Figures 1C, lanes 3-4, and 1E). Furthermore, when combined with imatinib, ABT-199 significantly enhanced imatinib-induced apoptosis in KCL22 (Figure 1C, compare lanes 2 and 4 with lane 6) but not in K562 or KYO1 cells (Figures 1B & 1D, compare lanes 2 and 4 with lane 6). Together, our data demonstrate that while CML cell lines exhibit differential sensitivity to single agent ABT-199, combination with imatinib can enhance the ability of ABT-199 to induce apoptosis in cell lines that express high levels of BCL2.

**Figure 2:**
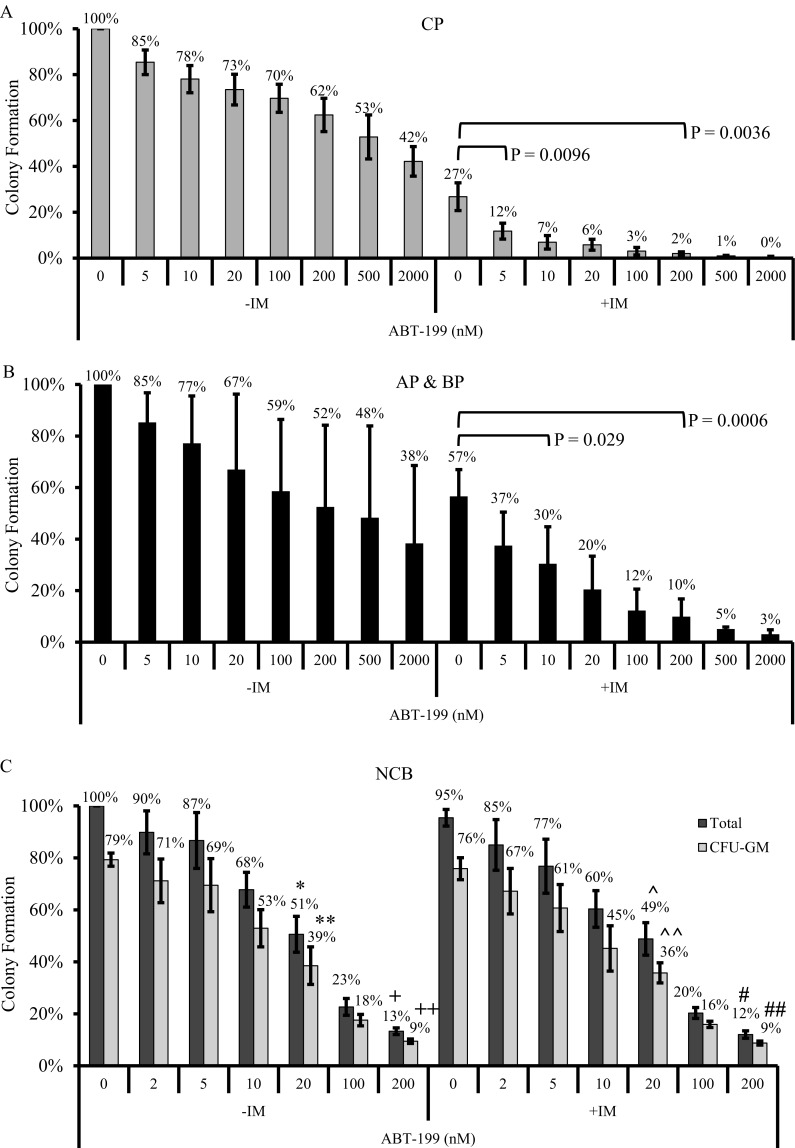
Colony formation assays were used to evaluate the effectiveness of ABT-199, as a single agent (-IM) or in combination with 2 uM imatinib (+IM), against both CML and normal cord blood (NCB) progenitors Results of the colony formation assays for (A) CP CML progenitors (n=4), (B) combined AP and BP CML progenitors (n=4), and (C) normal cord blood (NCB) progenitors (n=3). For NCB, both total and CFU-GM populations are shown. For (A) and (B), colony formation for each sample was calculated as a percentage of the total number of colony counted from the DMSO control. For (C), colony formation for each sample was calculated as a percentage of the number of colonies counted from the DMSO control of total population. Results are given as the mean +/− s.e.m. The P values were based on Student's *t* test. For (C), the P value for each of the indicated total or CFU-GM population was calculated by comparing to its corresponding DMSO control sample: *P= 0.00644, **P= 0.012, ^+^P= 7.3 × 10^−5^ and ^++^P= 5 × 10^−5^. Additionally, the following P value in (C) for each of the indicated total or CFU-GM population was calculated by comparison to the corresponding imatinib only treatment sample: ^^^P= 0.00146, ^^^^P= 7 × 10^−5^, ^#^P= 6.1 × 10^−5^, ^##^P= 1.9 × 10^−4^.

Since CML progenitors have recently been described to be particularly dependent on BCL2 for survival and maintenance [4, 5], we evaluated the effectiveness of ABT-199 in reducing the viability of primary CD34^+^ progenitors from patients in both early [chronic phase (CP)], and advanced stage [accelerated (AP)/blast phase (BP)] CML. At the same time, we also assessed the cytotoxic effect of ABT-199 on normal cord blood (NCB) progenitors. We evaluated the IC*_50_* and IC*_90_* of ABT-199 by colony formation assay (CFA), and used a broad concentration range of ABT-199 (0-2uM). The concentration of imatinib used was 2uM, which is in line with the plasma concentrations achievable in patients with CML [20]. For CP CML progenitors, imatinib potently reduced their average viability by 73% (Figure 2A). Compared to imatinib, ABT-199 had a modest effect on CP CML progenitors with an average IC*_50_* of 500nM (Figure 2A). The IC*_90_* was not achieved at the maximum concentration tested (2uM). However, when ABT-199 was combined with imatinib, the IC*_90_* was achieved at 5nM ABT-199, representing a 2-log improvement in efficacy compared to ABT-199 alone (Figure 2A). As for advanced stage CML progenitors, imatinib reduced their average viability by 43% (Figure 2B). Similar to CP progenitors, ABT-199 also had a modest effect on advanced stage CML progenitors with an average IC*_50_* of 500nM (Figure 2B). IC90 was not achieved at the maximum concentration tested (2uM). However, when ABT-199 was combined with imatinib, the viability of advanced stage CML progenitors was again significantly reduced with an average IC90 of 200nM ABT-199 (Figure 2B).

For NCB progenitors, imatinib had minimal effects on viability (Figure 2C). ABT-199, with or without imatinib, significantly reduced the viability of the total population of NCB progenitors, with average IC*_50_* and IC*_90_* values of 20nM and 200nM respectively (Figure 2C). It has been reported that for a given drug, the IC*_90_* for the CFU-GM (colony forming unit-granulocyte and macrophage) population of NCB progenitors is more predictive of the maximum tolerated dosage (MTD) than the IC*_50_* value [21]. We, therefore, assessed the effect of ABT-199, as a single agent or in combination with imatinib, on the viability of the CFU-GM population among NCB progenitors. We found that the average IC*_50_* and IC*_90_* values for ABT-199 were 20nM and 200nM respectively (Figure 2C). Thus, our results suggest that the MTD of ABT-199 for normal progenitors is 200nM.

**Figure 3:**
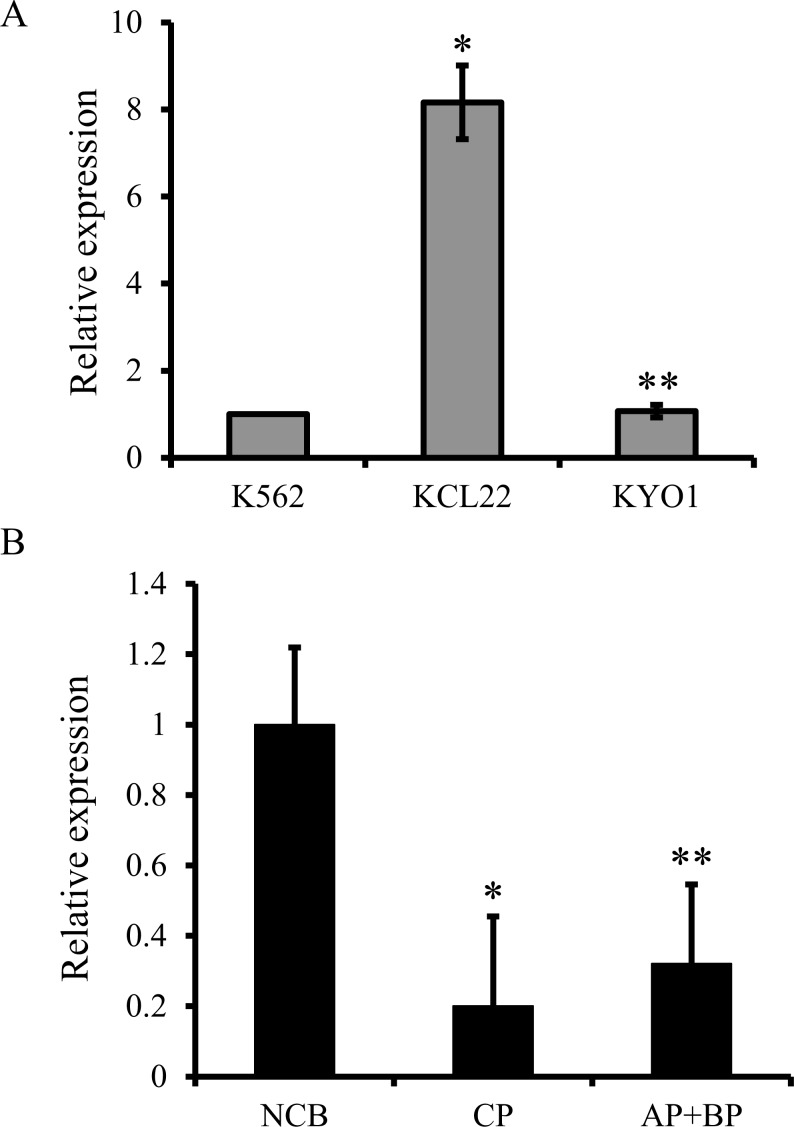
Real-time quantitative PCR assessment of the relative BCL2 mRNA expression levels in CML cell lines and primary progenitors (A) The relative BCL2 mRNA expression levels of three CML cell lines are shown. Relative expression is plotted as the ratio of the HPRT-normalized BCL2 mRNA expression level of a given cell line to that of K562. Results are given as the mean +/− s.e.m. (n=3). * P= 0.0046, **P= 0.49 (Student's *t* test). (B) The relative BCL2 mRNA expression levels for normal cord blood progenitors (NCB, n=3), CP CML progenitors (CP, n=4) and combined AP and BP CML progenitors (AP+BP, n=4). Relative expression is the ratio of the HPRT-normalized BCL2 mRNA expression level of a given sample to that of NCB. Results are given as the mean +/− s.e.m. * P= 0.0026, **P= 0.0022 (Student's *t* test).

Given that NCB progenitors were more sensitive to ABT-199 than CML progenitors, we determined if BCL2 levels were higher in the former, since high BCL2 expression levels predict ABT-199-sensitivity [15]. First, in CML cell lines, we confirmed the positive correlation between ABT-199-sensitivity and BCL2 expression at both the protein (Figure 1) and mRNA (Figure 3A) levels. Next, we observed a three- to five-fold greater expression of BCL2 mRNA in NCB progenitors compared to early and advanced stage CML progenitors (Figure 3B), a finding that may underlie the relative senstivity of NCB progenitors to ABT-199.

## DISCUSSION

We find that in CML and NCB progenitors, BCL2 expression levels predict sensitivity to the BCL2 antagonist, ABT-199, and mirror the findings in other human malignancies. Also, while ABT-199 alone had a modest effect on CML progenitors, combination therapy with imatinib enhanced ABT-199's inhibitory effects on both early and advanced stage CML progenitors by at least 13- and 5-fold respectively at the NCB IC_90_ of 200nM (Figure 2). Importantly, while our findings in NCB progenitors explain the dose-limiting hematologic toxicities observed in ABT-199-treated patients [22, 23], our results also predict that the combination of ABT-199 and imatinib may allow ABT-199 to be used at a concentration which would not harm normal progenitors.

## METHODS

### Ethics Statement

Investigation has been conducted in accordance with the ethical standards and according to the Declaration of Helsinki and according to national and international guidelines and has been approved by the authors' institutional review board.

### Cord blood and patient samples

CML samples were obtained from the Singapore General Hospital (Singapore). Patient informed consent was obtained under approved institutional review board (IRB) procedures. Cord blood samples were purchased from the Singapore Cord Blood Bank (Singapore).

### CD34^+^ isolation and colony formation assay

CD34^+^ cells were prepared and cultured as described [24]. CD34^+^ cells were subjected to drug treatment for 72 hours in StemPro (with supplements [24]; Life Technologies, Carlsbad, CA) before they were seeded, 1000 cells per 35mm plate (in duplicate), in drug-free methylcellulose (H4434; Stemcell Technologies, Vancouver, Canada), and colonies counted after 10-12 days.

### Cell lines, cell culture and chemicals

KCL22, K562 and KYO1 CML cell lines were cultured as described [7]. Imatinib was obtained from tablets as described [7]. ABT-199 was purchased from ChemieTek (Indianapolis, IN).

### Immunoblot, ELISA-based DNA fragmentation assay, RNA extraction, cDNA synthesis and real-time qPCR

The procedures, reagents, and primer sequences have been described previously [5, 7].

## References

[1] Druker BJ, Guilhot F, O'Brien SG, Gathmann I, Kantarjian H, Gattermann N, Deininger MW, Silver RT, Goldman JM, Stone RM, Cervantes F, Hochhaus A, Powell BL, Gabrilove JL, Rousselot P, Reiffers J (2006). Five-year follow-up of patients receiving imatinib for chronic myeloid leukemia. The New England journal of medicine.

[2] Bhatia R, Holtz M, Niu N, Gray R, Snyder DS, Sawyers CL, Arber DA, Slovak ML, Forman SJ (2003). Persistence of malignant hematopoietic progenitors in chronic myelogenous leukemia patients in complete cytogenetic remission following imatinib mesylate treatment. Blood.

[3] Copland M, Hamilton A, Elrick LJ, Baird JW, Allan EK, Jordanides N, Barow M, Mountford JC, Holyoake TL (2006). Dasatinib (BMS-354825) targets an earlier progenitor population than imatinib in primary CML but does not eliminate the quiescent fraction. Blood.

[4] Lagadinou ED, Sach A, Callahan K, Rossi RM, Neering SJ, Minhajuddin M, Ashton JM, Pei S, Grose V, O'Dwyer KM, Liesveld JL, Brookes PS, Becker MW, Jordan CT (2013). BCL-2 inhibition targets oxidative phosphorylation and selectively eradicates quiescent human leukemia stem cells. Cell stem cell.

[5] Goff DJ, Recart AC, Sadarangani A, Chun HJ, Barrett CL, Krajewska M, Leu H, Low-Marchelli J, Ma W, Shih AY, Wei J, Zhai D, Geron I, Pu M, Bao L, Chuang R (2013). A Pan-BCL2 inhibitor renders bone-marrow-resident human leukemia stem cells sensitive to tyrosine kinase inhibition. Cell stem cell.

[6] Kuroda J, Kimura S, Andreeff M, Ashihara E, Kamitsuji Y, Yokota A, Kawata E, Takeuchi M, Tanaka R, Murotani Y, Matsumoto Y, Tanaka H, Strasser A, Taniwaki M, Maekawa T (2008). ABT-737 is a useful component of combinatory chemotherapies for chronic myeloid leukaemias with diverse drug-resistance mechanisms. British journal of haematology.

[7] Ng KP, Hillmer AM, Chuah CT, Juan WC, Ko TK, Teo AS, Ariyaratne PN, Takahashi N, Sawada K, Fei Y, Soh S, Lee WH, Huang JW, Allen JC, Woo XY, Nagarajan N (2012). A common BIM deletion polymorphism mediates intrinsic resistance and inferior responses to tyrosine kinase inhibitors in cancer. Nature medicine.

[8] Airiau K, Mahon FX, Josselin M, Jeanneteau M, Turcq B, Belloc F (2012). ABT-737 increases tyrosine kinase inhibitor-induced apoptosis in chronic myeloid leukemia cells through XIAP downregulation and sensitizes CD34(+) CD38(-) population to imatinib. Experimental hematology.

[9] Mak DH, Wang RY, Schober WD, Konopleva M, Cortes J, Kantarjian H, Andreeff M, Carter BZ (2012). Activation of apoptosis signaling eliminates CD34+ progenitor cells in blast crisis CML independent of response to tyrosine kinase inhibitors. Leukemia.

[10] Oltersdorf T, Elmore SW, Shoemaker AR, Armstrong RC, Augeri DJ, Belli BA, Bruncko M, Deckwerth TL, Dinges J, Hajduk PJ, Joseph MK, Kitada S, Korsmeyer SJ, Kunzer AR, Letai A, Li C (2005). An inhibitor of Bcl-2 family proteins induces regression of solid tumours. Nature.

[11] Tse C, Shoemaker AR, Adickes J, Anderson MG, Chen J, Jin S, Johnson EF, Marsh KC, Mitten MJ, Nimmer P, Roberts L, Tahir SK, Xiao Y, Yang X, Zhang H, Fesik S (2008). ABT-263: a potent and orally bioavailable Bcl-2 family inhibitor. Cancer research.

[12] Wilson WH, O'Connor OA, Czuczman MS, LaCasce AS, Gerecitano JF, Leonard JP, Tulpule A, Dunleavy K, Xiong H, Chiu YL, Cui Y, Busman T, Elmore SW, Rosenberg SH, Krivoshik AP, Enschede SH (2010). Navitoclax, a targeted high-affinity inhibitor of BCL-2, in lymphoid malignancies: a phase 1 dose-escalation study of safety, pharmacokinetics, pharmacodynamics, and antitumour activity. The lancet oncology.

[13] Zhang H, Nimmer PM, Tahir SK, Chen J, Fryer RM, Hahn KR, Iciek LA, Morgan SJ, Nasarre MC, Nelson R, Preusser LC, Reinhart GA, Smith ML, Rosenberg SH, Elmore SW, Tse C (2007). Bcl-2 family proteins are essential for platelet survival. Cell death and differentiation.

[14] Mason KD, Carpinelli MR, Fletcher JI, Collinge JE, Hilton AA, Ellis S, Kelly PN, Ekert PG, Metcalf D, Roberts AW, Huang DC, Kile BT (2007). Programmed anuclear cell death delimits platelet life span. Cell.

[15] Souers AJ, Leverson JD, Boghaert ER, Ackler SL, Catron ND, Chen J, Dayton BD, Ding H, Enschede SH, Fairbrother WJ, Huang DC, Hymowitz SG, Jin S, Khaw SL, Kovar PJ, Lam LT (2013). ABT-199, a potent and selective BCL-2 inhibitor, achieves antitumor activity while sparing platelets. Nature medicine.

[16] Horita M, Andreu EJ, Benito A, Arbona C, Sanz C, Benet I, Prosper F, Fernandez-Luna JL (2000). Blockade of the BCR-ABL1 kinase activity induces apoptosis of chronic myelogenous leukemia cells by suppressing signal transducer and activator of transcription 5-dependent expression of Bcl-xL. The Journal of experimental medicine.

[17] Aichberger KJ, Mayerhofer M, Krauth MT, Skvara H, Florian S, Sonneck K, Akgul C, Derdak S, Pickl WF, Wacheck V, Selzer E, Monia BP, Moriggl R, Valent P, Sillaber C (2005). Identification of mcl-1 as a BCR/ABL-dependent target in chronic myeloid leukemia (CML): evidence for cooperative antileukemic effects of imatinib and mcl-1 antisense oligonucleotides. Blood.

[18] Sanchez-Garcia I, Grutz G (1995). Tumorigenic activity of the BCR-ABL1 oncogenes is mediated by BCL2. Proc Natl Acad Sci USA.

[19] Mahon FX, Deininger MW, Schultheis B, Chabrol J, Reiffers J, Goldman JM, Melo JV (2000). Selection and characterization of BCR-ABL1 positive cell lines with differential sensitivity to the tyrosine kinase inhibitor STI571: diverse mechanisms of resistance. Blood.

[20] Peng B, Hayes M, Resta D, Racine-Poon A, Druker BJ, Talpaz M, Sawyers CL, Rosamilia M, Ford J, Lloyd P, Capdeville R (2004). Pharmacokinetics and pharmacodynamics of imatinib in a phase I trial with chronic myeloid leukemia patients. Journal of clinical oncology: official journal of the American Society of Clinical Oncology.

[21] Pessina A, Albella B, Bayo M, Bueren J, Brantom P, Casati S, Croera C, Gagliardi G, Foti P, Parchment R, Parent-Massin D, Schoeters G, Sibiril Y, Van Den Heuvel R, Gribaldo L (2003). Application of the CFU-GM assay to predict acute drug-induced neutropenia: an international blind trial to validate a prediction model for the maximum tolerated dose (MTD) of myelosuppressive xenobiotics. Toxicological sciences: an official journal of the Society of Toxicology.

[22] Roberts A, Davids M, Page J, Kahl B, Wierda W, Miller T, Gerecitano J, Kipps T, Anderson M, Huang D, Darden D, Gressick L, Nolan C, Yang J, Busman T, Graham A (2013). The BCL-2 inhibitor ABT-199 (GDC-0199) is active and well-tolerated in ultra high-risk relapsed/refractory chronic lymphocytic leukemia (CLL) [abstract]. Haematologica.

[23] Davids M, Seymour J, Gerecitano J, Kahl B, Pagel J, Wierda W, Anderson M, Darden D, Nolan C, Gressick L, Yang J, Chyla B, Busman T, Graham A, Cern E, Enschede S (2013). The BCL-2 inhibitor ABT-199 (GDC-0199) is active and well-tolerated in patients with relapsed/refractory mantle cell lymphoma and other non-hodgkin lymphomas [abstract]. Haematologica.

[24] Lim S, Saw TY, Zhang M, Janes MR, Nacro K, Hill J, Lim AQ, Chang CT, Fruman DA, Rizzieri DA, Tan SY, Fan H, Chuah CT, Ong ST (2013). Targeting of the MNK-eIF4E axis in blast crisis chronic myeloid leukemia inhibits leukemia stem cell function. Proc Natl Acad Sci USA.

